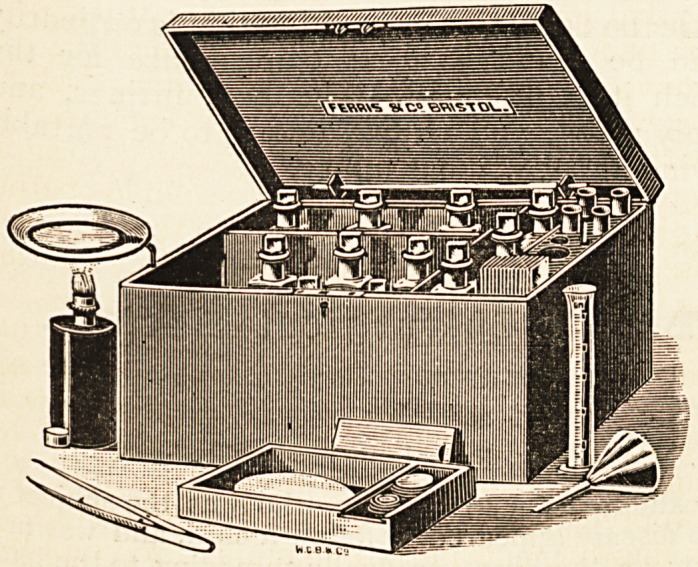# Notes on Preparations for the Sick

**Published:** 1891-06

**Authors:** 


### Notes on Preparations for the Sick.

**Malted Milk. Dry Extract of Malt.**—MALTED MILK Co., London.

**Malted Milk** is another of the many useful additions to the dietary of the sick. It has only to be dissolved in warm water; no cooking is required, no milk need be added; it is partially predigested, and it does not contain starch. Its flavour is excellent. For delicate infants, who cannot digest ordinary milk, and in many conditions of acute disease or weak digestion, this easily-assimilated and nutritious food should be invaluable.

The **Dry Extract of Malt** has the following advantages over the liquid extracts. It will not change or ferment in hot weather, and can be carried or kept on hand with greater safety and convenience. It can also be taken with greater ease, dissolved in quantities to suit the taste, and may be used in many ways not possible with a liquid extract. It is more palatable to the sick and to children, and has less the appearance of medicine. It contains all the natural salts of the grain in a soluble form combined with the sugar of the malt. It may be dissolved in milk or water, or mingled with any food. It will take the place of ordinary sugar in any mixture or emulsion.

**Granular Effervescent Exalgine (gr. ij. in ʒj). Granular Effervescent Sodæ Sulph. Granular Effervescent Magnes. Sulph.**—G. T. Turner, Clifton.

We have received the above preparations of our old and well-tried Epsom and Glauber's salts, and of the more modern analgesic exalgine. The saline aperients in effervescing form are in every-day use, and need no commendation; the sulphates are likely to compete successfully with the effervescing citrates.

The **Effervescing Exalgine** is a useful preparation, and may be safely given in teaspoonful doses for several successive hours until the wished-for relief is secured.

---

**Upjohn's Friable Pills.**—The Upjohn Pill and Granule Co., Kalamazoo, Mich., U.S.A.

Messrs. Hodder & Co., the local agents, have sent us samples of these pills, concerning which the manufacturers write:

"To overcome the objections to both gelatine capsules and coated mass pills, and produce a means of administration which insures the liberation and solution of the powdered drugs while still in the stomach, and at the same time entirely obviate the tendency to become hard and worthless by age, has been a matter that has received a vast amount of personal detailed attention and study from us. To accomplish our aim, we have devised a method of pill-making which entirely does away with the necessity of either excipient or moisture as a part of the pill; the dry powdered drug is thoroughly protected by a thin and extremely soluble coating of sugar which rapidly dissolves when taken into the stomach, leaving the enclosed powder in the same condition for solution and absorption as when administered in powdered form."

Pill-making has reached such a degree of perfection that it is difficult to find sufficient scope for utilising all the excellent materials provided. In fact, it will be necessary to cultivate prophylactic treatment, as for instance that for influenza, and administer pills to those in good health. No one could fail to appreciate the excellence of manufacture of these, the latest improvment in pill manufacture, and we have found them to be as efficient as they are inviting. Their friable and powdery condition is easily demonstrated by simply crushing them beneath the thumb. The ingredients of a pill thus made must be ready for immediate absorption upon their arrival in the stomach.

---

**Molfa Soap.**—Dinneford & Co., London.

**Juvenia Toilet Soap. Juvenia Cream.**—F. S. Cleaver & Sons, London.

The manufacture of **Molfa Soap** is carried on under the supervision of Dr. B. H. Paul, great care being taken to regulate its composition and — by carefully excluding an excess of alkali, essential oils and colouring matter, which make even good soaps injurious to the skin—to produce a perfect soap. We think the manufacturers have fully accomplished the object in view.

The makers of the **Juvenia Soap** and **Cream** have also been most anxious to reach perfection, and think they have succeeded. The preparations are excellent, requiring only to be known to win favour, it being intended that the soap shall be used by day, and the cream at night.

---

**Box containing Complete Apparatus for Clinical Detection of Micro-organisms in Blood, Sputum, and Urine.**

Dr. J. Michell Clarke writes: "It has occurred to me in the course of ordinary work that it would be very convenient —especially for those engaged in hospital practice —to have in a small compass and handy form all the materials necessary for the staining of micro-organisms in the excretions and in the blood. When the reagents are not thus gathered together, there is often much time wasted in finding the particular ones required for a given investigation.

"At my suggestion, Messrs. Ferris & Co., of Bristol, have devised a small box, which contains, conveniently arranged, the materials most commonly required. The box is of small size, so as to be easily carried about.

"Briefly, it contains the following: Ziehl-Neelsen fluid, with solution of nitric acid for decolourising, and methylene blue solution for ground stain (tubercle bacilli); the materials for making the Weigert-Ehrlich fluid, which requires to be freshly made, and is one of the most generally useful staining fluids; Gram's solution of iodine and iodide of potash for use with the Weigert-Ehrlich fluid, and a solution of acetic acid (1—5) for use with the same staining fluid for the examination of blood after Günther's method; the methylene-blue is also very useful as a general stain for micro-organisms. The box also contains bottles of distilled water, absolute alcohol, and Canada balsam dissolved in xylol; vesuvin and Bismarck-brown in powder, to be dissolved in water as required for use; needles, pipettes, glass rod, forceps, glass measure of 10 c.c. capacity; funnel and filter papers, watch-glasses, test tubes, microscopic slides, and cover-glasses. There is a spirit-lamp and a box with close-fitting cover, in which the cover-glasses can be left to dry. A support for carrying a watch-glass above the lamp can be pushed out when the box is open. There is an extra bottle, which has been purposely left empty for the addition of any particular fluid that the owner may desire in addition to those enumerated above.

"I think that the box contains everything that is ordinarily required, and will be found a great convenience for the purposes for which it is intended. The box, fittings, and bottles are strongly made, and arranged so as to be portable and to bear carrying about without injury."

## Figures and Tables

**Figure f1:**